# Improvement of the Thermal Insulation Performance of Silica Aerogel by Proper Heat Treatment: Microporous Structures Changes and Pyrolysis Mechanism

**DOI:** 10.3390/gels8030141

**Published:** 2022-02-23

**Authors:** Zhiyi Lun, Lunlun Gong, Zhongxin Zhang, Yurui Deng, Yong Zhou, Yuelei Pan, Xudong Cheng

**Affiliations:** State Key Laboratory of Fire Science, University of Science and Technology of China, Hefei 230027, China; lunzhiyi@mail.ustc.edu.cn (Z.L.); gongll@ustc.edu.cn (L.G.); zhxinzh@mail.ustc.edu.cn (Z.Z.); dyr@mail.ustc.edu.cn (Y.D.); yongz@ustc.edu.cn (Y.Z.)

**Keywords:** thermal insulation performance, surface groups, heat treatment, pyrolysis, structural modeling

## Abstract

A simple heat treatment method was used to optimize the three-dimensional network structure of the hydrophobic aerogel, and during the heat treatment process at 200–1000 °C, the thermal conductivity of the aerogel reached the lowest to 0.02240 W/m·K between 250 °C and 300 °C, which was mainly due to the optimization of microstructure and pyrolysis of surface groups. Further Fluent heat-transfer simulation also confirmed the above results. Synchrotron vacuum ultraviolet photoionization mass spectrometry (SVUV-PIMS) was used to finely measure the pyrolysis process of aerogels, and the pyrolysis process of aerogel was divided into four stages. (I) Until 419 °C, as the temperature continued to rise, surface methyl groups were oxidized to form hydroxyl. (II) As the temperature reached to 232 °C, the oxidation proceeded. In addition, inside the aerogel, because of lacking oxygen, the reaction produced CH_4_ and C–Si bonds would form. (III) After 283 °C, Si–OH groups began to condense to form Si–O–Si, which optimized the three-dimensional network structures to be beneficial to improve the thermal insulation performance of silica aerogel. (IV) When it reached 547 °C, the chemical reaction was terminated, and all the primary particles gradually fused into secondary particles and sintered to form clusters.

## 1. Introduction

With the increasing demand for energy utilization [1,2,3] and the importance of thermal safety, the application range of thermal insulation materials has gradually expanded. Insulation materials with excellent performance can not only significantly reduce the heat loss in some scenes [4,5,6], but also control the temperature in certain technological processes [7] and improve the stability of high-temperatures [8], which plays an important role in industry and commerce [9]. As a material with excellent performance, silica aerogel has extremely low density and thermal conductivity [10,11]. Therefore, its application potential in many fields, especially thermal insulation materials [12], has attracted much attention. Aerogel is composed of a nano-porous network framework [13]. The mean free path of air molecules is larger than the typical pore size. The heat transfer process requires numerous pores, resulting in aerogels’ thermal conductivity significantly lower than that of air. The resulting aerogel films [14], fibers [15], boards [16], blankets [17], and other products have been widely used in thermal insulation systems in construction, petroleum, chemical, and other fields.

The preparation process of aerogels has been continuously matured and perfected with the continuous in-depth research on aerogels, and how to further reduce thermal conductivity of aerogels has become a core issue for researchers. Li et al. [18] confirmed that the thermal properties of the aerogels can be tailored by adjusting the concentration and types of acid-base catalysts. The study found that with the concentration of alkaline catalyst increasing, the thermal conductivity can be reduced by up to 66% (0.027 vs. 0.08 W/m·K), which caused by the reduction in cluster size and the richness and uniformity of pores. Zhou et al. [19] and Ul Haq et al. [20] have used different sol solubility to impregnate the glass fiber mat to study the thermal insulation properties of the aerogel composite insulation material after preparation. Pan et al. [21] synthesized hybrid silica with good thermal isolation performances by co-precursor. The study has found that selecting suitable silicon precursors for combination can effectively improve the three-dimensional orientation structure of aerogels, thereby enhancing the thermal insulation properties. According to Iswar et al. [22], aging plays an important role to strengthen the gel network which can help to obtain silica aerogel with low thermal conductivity. Most of the research has focused on adjusting the process flow during the preparation process to optimize the thermal insulation performance of aerogels, but few researchers paid attention the improvement and adjustment of thermal insulation performance after the aerogel is prepared, which is considered to be an effective way to further improve the actual use effect of aerogels.

In this study, a simpler heat treatment method was used to optimize the three-dimensional network structure of hydrophobic silica aerogel, thus improving the thermal insulation performance of the aerogel. The physicochemical changes of the aerogels are studied in depth, and the reasons for the influence of the pyrolysis process on the thermal conductivity of the aerogels are analyzed by comparing the changes in structure and surface groups. Finally, the proper heat treatment temperatures were obtained to minimize the thermal conductivity of the hydrophobic silica aerogel.

## 2. Results and Discussion

### 2.1. Thermal Insulation Performance and Micro Morphology

**Figure 1:**
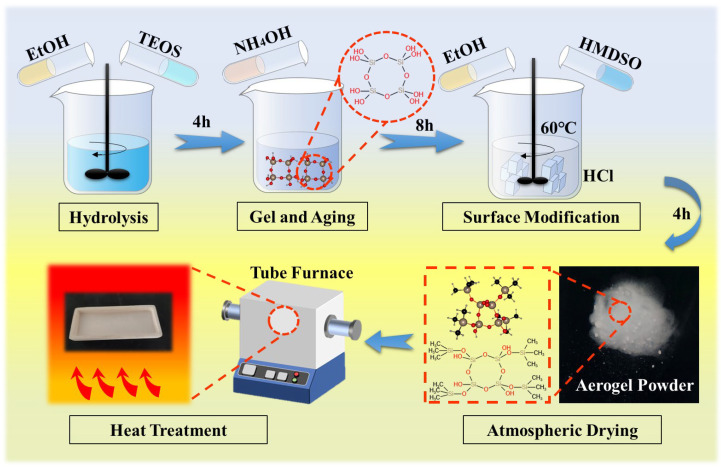
Preparation process and heat treatment optimization of hydrophobic silica aerogel.

Hydrophobic silica aerogel was successfully synthesized [23] at ambient pressure through sol-gel method and surface modification by hexamethyldisiloxane (HMDSO) using tetraethyl orthosilicate as precursor, as shown in Figure 1. In brief, TEOS ethanol solution was hydrolyzed under acidic conditions, and then NH_4_OH was added to adjust the pH value to obtain gel. After the surface modification through HMDSO, the hydrophobic silica aerogel powder could be obtained by atmospheric drying. Finally, the thermal insulation of the aerogel was optimized by suitable heat treatment. After heat treatment, the thermal properties of silica aerogel have changed. As shown in Table 1, heat-treated samples separated by 50 °C also participated in the inspection. Silica aerogel treated by high-temperatures would shrink (Figure S1), and with the increase in the heat treatment temperature, the thermal conductivity exhibited significantly decrease to the lowest of 0.02242 W/m·K at 300 °C and then increase from 350 °C to 1000 °C. In order to study the mechanism that the thermal insulation performance of silica aerogel changes with the heat treatment temperature, the influence of temperature on the hydrophobic group and structure had been analyzed. Through N_2_ desorption-adsorption isotherms (Figure S2), the overall structure of silica aerogel could be characterized. The type IV isotherms changed [24], with the heat treatment temperature increased, proving that the mesopores of the silica aerogel gradually collapse and disappear after 800 °C.

**Figure 2:**
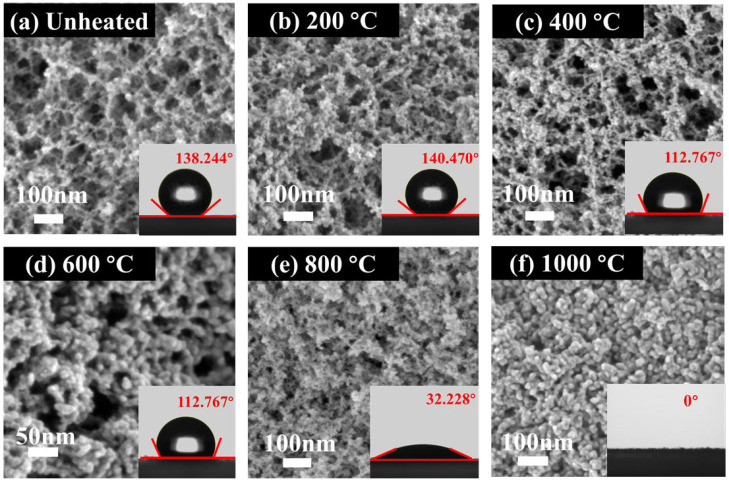
SEM images of silica aerogel treated at different heat treatment temperatures (**a**) Unheated, (**b**) 200 °C, (**c**) 400 °C, (**d**) 600 °C, (**e**) 800 °C, (**f**) 1000 °C.

The SEM photos of both virgin and heated samples under over 200 °C, 400 °C, 600 °C, 800 °C, and 1000 °C are shown in Figure 2a–f. The virgin sample had a similar nanostructure with the samples treated under 200 °C to 400 °C, and both of them were highly porous and homogeneous. As the temperature increased, the pyrolysis process of the aerogel sample continued to advance, and the surface groups changed. Most of secondary particles aggregated [25] under 500 °C to 600 °C with the breakdown of the uniform pore structure. For the heat-treated sample under 600 °C, the larger particles had coalesced to form from secondary particles. Finally, as the secondary particles continued to polymerize, larger particle clusters were formed, the three-dimensional network structure was destroyed, and the number of macropores increased sharply for the sample under 1000 °C heat treated. The previously prepared silica aerogels had excellent hydrophobic property, and during surface modification, methyl was attached to the surface which contributes to hydrophobicity of the silica aerogel [19]. As shown in Figure 2a–f, with the pyrolysis of the aerogel samples, its hydrophobic angle rise slightly from 138.244° to 140.470° and then drop sharply, which caused by the increased of the number of methyl groups.

### 2.2. Surface Groups and BET Analysis

**Figure 3:**
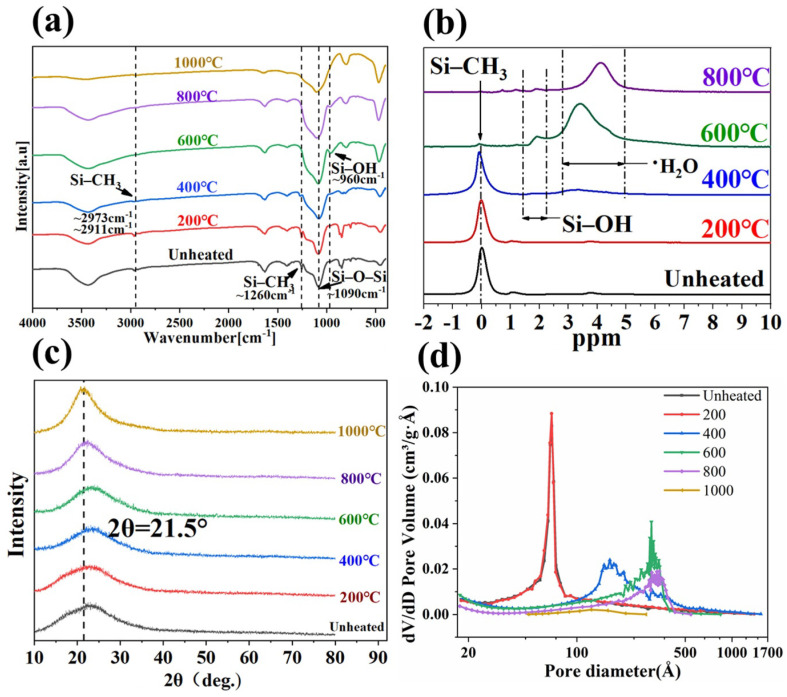
(**a**) FT-IR spectra, (**b**) ^1^H NMR spectra, (**c**) XRD patterns and (**d**) Pore size distributions of the silica aerogel samples treated at different temperature.

In order to determine the changes in surface groups of silica aerogel samples during pyrolysis, FTIR tests were conducted. As shown in Figure 3a, for silica aerogels after heat treatment at different temperatures, in the spectrum, peak of ~960 cm^−1^ were observed for the sample heat treated at 400 °C, which was due to the stretching vibration of Si–OH [26] and it disappeared after 800 °C. The peak of ~2973 cm^−1^, ~2911 cm^−1^ and ~1260 cm^−1^ from the stretching, bending and deformation vibration of C–H bonds of Si–CH_3_ groups, respectively [27,28], gradually became weaker and eventually could not be observed after 800 °C. The changes of peaks indicated that when the silica aerogel was pyrolyzed, Si–CH_3_ was oxidized and Si–OH was produced. In addition, during the pyrolysis process, as the temperature increased, the peak signal of ~1090 cm^−1^ continued to increase, which proved that Si–O–Si was continuously generated. The solid nuclear magnetic test also showed similar results. As can be seen from Figure 3b, the untreated and heat-treated under 200 °C and 400 °C silica aerogel had a sharp peak at 0 ppm, indicating the methyl group attached to Si. After heat treatment at 600 °C, the peak at 0 ppm disappeared, and the peak at 2 ppm increased, indicating that during 400 °C to 600 °C, most of the methyl groups attached to Si were oxidized to produce hydroxyl groups. The results of the ^1^H NMR spectra of silica aerogel was consistent with the previous study [29].

In the process of heat treatment, the structure and surface groups of silica aerogel would change, but there was no crystal structure appeared, which could be derived from X-ray diffraction analysis in Figure 3c. The broad peak centered at 22° proved that the amorphous structure of silica aerogels was still retained after heat treatment. At the same time, it also eliminated the possibility of producing compounds that contribute to the thermal conductivity during the pyrolysis process. The pore diameter distribution characteristic was also one of the important aspects reflecting the thermal conductivity. As shown in Figure 3d, the pore size distribution of the samples that were not been affected by heat treatment was between 50–100 Å, and as the heat treatment temperature increased, the pore size distribution range gradually expands to between 100 and 500 Å. At the same time, when the heat treatment temperature continued to rise to 400 °C, the BET surface area continued to rise from 878 m^2^/g to 993 m^2^/g, as shown in Table 2, and pores volume also reached a peak of 4.75 cm^3^/g. As the heat treatment temperature continues to rise to 1000 °C, both BET surface area and pores volume rapidly drop to only 54 m^2^/g and 0.23 cm^3^/g.

### 2.3. XPS Analysis

**Figure 4:**
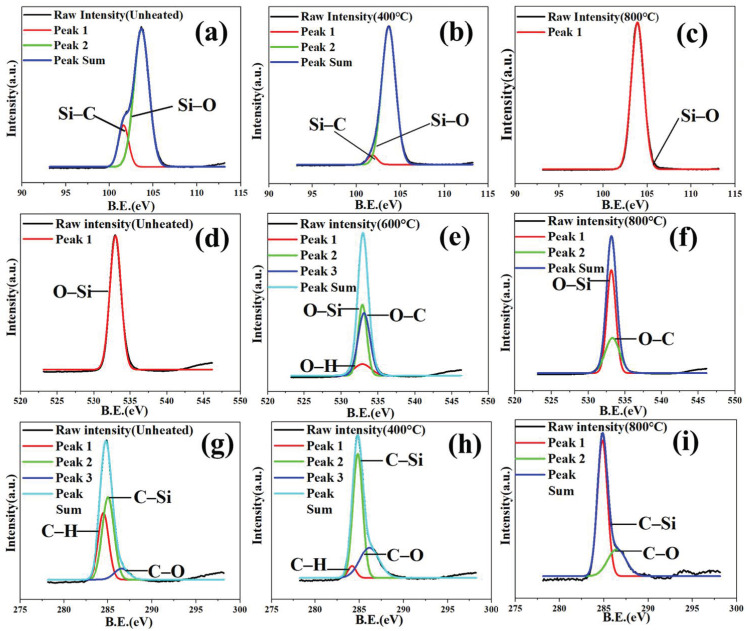
Si 2p spectra of the silica aerogel heat treated at different temperatures: (**a**) Unheated, (**b**) 400 °C and (**c**) 800 °C, respectively. O 1 s spectra of the silica aerogel heat treated at different temperatures: (**d**) Unheated, (**e**) 600 °C, and (**f**) 800 °C, respectively. C 1 s spectra of the silica aerogel heat treated at different temperatures: (**g**) Unheated, (**h**) 400 °C, and (**i**) 800 °C, respectively.

To study the changes in the components of silica aerogel during heat treatment, XPS experiments were performed, as shown in Table 3 and Figure 4. During the pyrolysis of silica aerogel, silicon would not become part of the gaseous component, so the content of Si in the samples remained constantly. With the increase in temperature, the content in Table 3 show that the atom molar ratio of Si/C kept increasing from 0.70 to 5.02 while the molar ratio of Si/O decreased from 0.53, firstly, and then increased to 0.47, which was consistent with the group reaction discussed above. Namely, the oxidation of the methyl group decreased the value of Si/O with a biggest drop to 0.40, and the generated hydroxyl group increased the value of Si/O with a maximum growth (17.5%). Then, the condensation of the hydroxyl group generated Si–O–Si, which increased the oxygen content from 44.28% to 64.07%. It was worth noting that even after high-temperature sintering at 1000 °C in the samples, the element C in the silica aerogel was still remained [30].

Peak-differentiating and imitating were conducted to further analyze the chemical composition of the samples [31]. As shown in Figure 4a, the Si 2p peak was divided into two peaks assigned to the Si–C and Si–O bond that locate at 101.7 eV and 103.7 eV. Compared with the untreated aerogel, the 101.7 eV (Si–C) signal of the sample heat-treated at 400 °C (Figure 4b) was weakened, while the sample heat-treated at 800 °C (Figure 4c) could only be fitted with the peak located at 103.7 eV (Si–O), which showed the changing process of the methyl group attached to Si. For the element O, as shown in Figure 4d, the spectrum of the original silica aerogel was only fitted with a single peak component O–Si at 532.9 eV, while three peak components could fit to the spectra of silica aerogel heat treatment 600 °C (Figure 4e), 532.9 eV for O–Si, 532.8 eV for O–C, and 532.86 eV for O–H, respectively, which proved that methyl was gradually oxidized to hydroxyl during heat treatment. However, the spectra of the silica aerogel heat treatment at 800 °C (Figure 4f) was fitted by just two peak components, 532.8 eV for O–C and 532.9 eV for O–Si. For the element C, as shown in Figure 4g, the peak of C 1 s could be divided to three peaks at 285.0 eV, 284.2 eV, and 286.2 eV, which could be indexed to C–Si, C–H, and C–O. As the heat treatment temperature rise, the signal of peak at 284.2 eV (C–H) decreases (Figure 4h) while the spectra of the silica aerogel heat treatment at 800 °C (Figure 4i) was fitted by just two peak components, 286.2 eV (C–O) and 285.0 eV (C–Si). The splitting results of O 1 s and C 1 s confirmed the element composition in Table 2. The methyl group was oxidized to hydroxyl even if the silica aerogel underwent high-temperature heat treatment, and the hydroxyl group condensed to form Si–O, but the element C still existed in the form of C–O and C–Si.

### 2.4. Pyrolysis Mechanism

**Figure 5:**
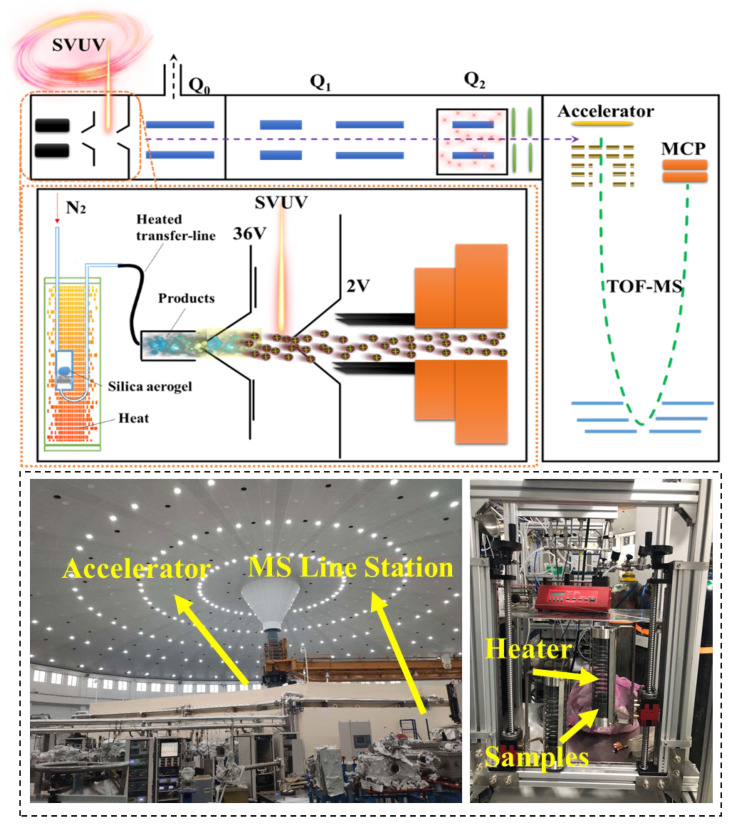
Experiment equipment of SVUV-PIMS analysis.

**Figure 6:**
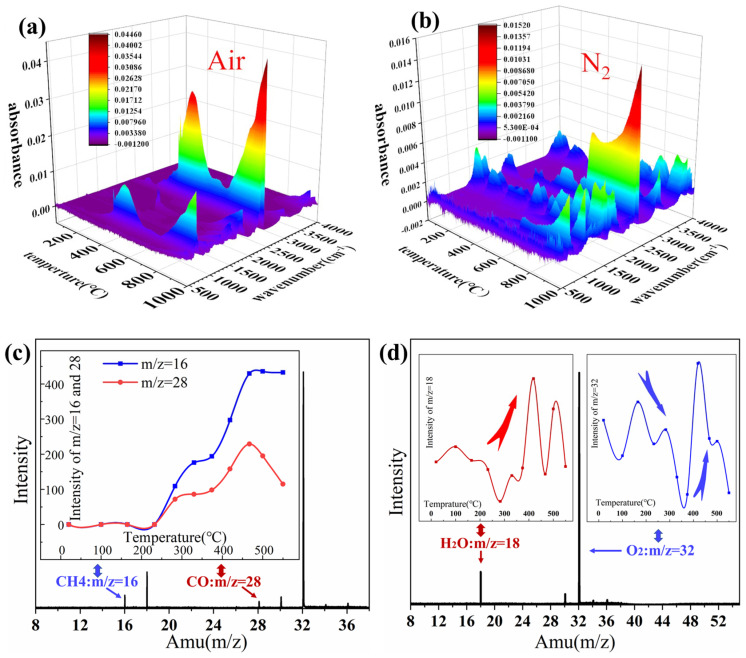
TG-IR spectra of the untreated silica aerogel under (**a**) air and (**b**) N_2_. (**c**) SVUV-PIMS of background and signal intensity trends of m/z = 18 and 32. (**d**) SVUV-PIMS at 450 °C and signal intensity trends of m/z = 16 and 28.

To study the reason why element C remained in the samples after high-temperature heat treatment and the pyrolysis reaction of aerogel at high-temperatures, TG-IR and synchrotron vacuum ultraviolet photoionization mass spectrometry (SVUV-PIMS) analysis was performed. The test principle of SVUV-PIMS and the equipment used were shown in Figure 5. The samples were heated up under nitrogen and oxygen conditions, respectively and the results were shown in Figure 6a,b. In the gas produced under N_2_ atmosphere, the observation of peaks around ~3000 cm^−1^ indicated that CH_4_ was produced with the heating process. Additionally, the peaks around 2150 cm^−1^ proved that small amount of CO was produced. In addition, it could be concluded that the peaks of 670 cm^−1^, 2350 cm^−1^, and 650 cm^−1^ mean that final products in the pyrolysis process of silica aerogel in air were CO_2_ and H_2_O, which both followed similar generation. SVUV-PIMS provided information on the pyrolysis substances of silica aerogel during the heating process, which could be used to analyze the gas products of the pyrolysis of silica aerogel. It could be concluded from the background signal graph that there was a certain amount of water and oxygen in the test environment, corresponding to m/z = 18 and m/z = 32. When the temperature continued to rise, the aerogel sample started to pyrolyze, which could be obtained from Figure 6c. The signal of m/z = 16 and m/z = 28 started to appear (Figure 6c), corresponding to CH_4_ and CO. Analyzing the mass spectrum data could be obtained that, in addition to the newly generated gas, the signal has also changed, so the graph of the signal intensity over time was used for further analysis. From the signal intensity of m/z = 16 and m/z = 28, under the condition of heating rate of 10 °C/min, it could be concluded that the temperature continued to rise to 232 °C, the aerogel samples began to pyrolyze to produce CO and CH_4_. Before 283 °C, some of the water originally contained in the sample was first released. After 283 °C, the aerogel samples began to pyrolyze and produce water (Figure 6d). During this process, comparing the signal diagram of m/z = 32, it could be concluded that the process of pyrolysis consumed oxygen form 165 °C to 359 °C.

**Figure 7:**
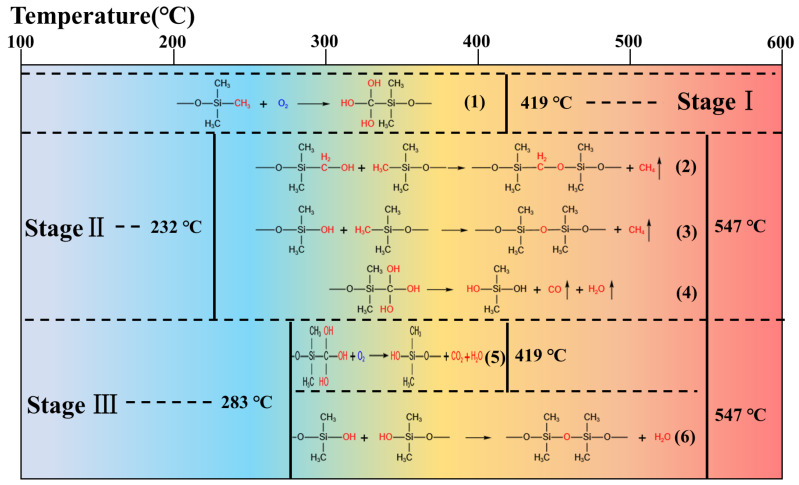
The chemical pyrolysis process of silica aerogel.

When the hydrophobic silica aerogels were heated in the air, a series of reactions would occur, as the surface and internal reactions would be different [32], and the whole process could be divided in four stages. The possible reactions at various stages of pyrolysis were showed in t following chemical formulas (Equations (1)–(6)) and Figure 7.


(1)

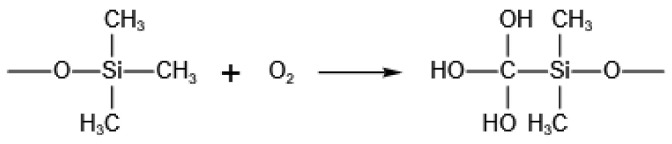




(2)

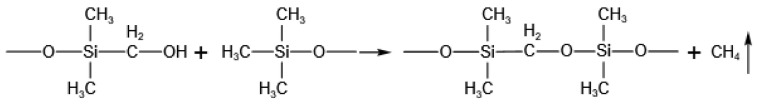




(3)

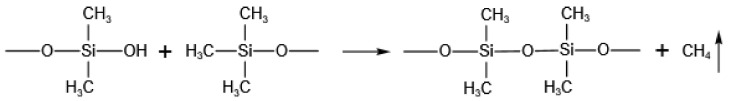




(4)

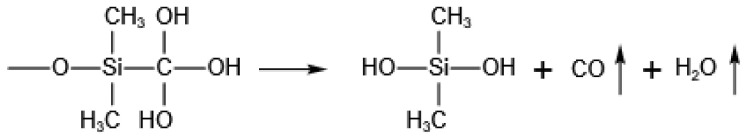




(5)

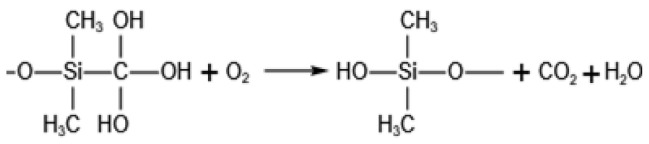




(6)

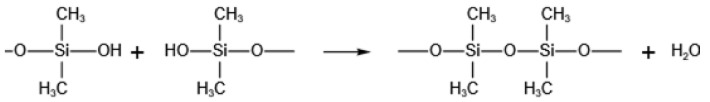



In stage I, until 419 °C, as the temperature continued to rise, the surface methyl groups were oxidized to form hydroxyl (Equation (1)). Stage II began after 232 °C. In the interior of silica aerogels, due to the lack of oxygen, the oxidation process was hindered. Related reactions and products could refer to the pyrolysis of silica aerogel under N_2_ conditions. Some methyl groups had not been oxidized, and they would react with hydroxyl groups to form CH_4_ (Equations (2) and (3)) and C–Si (Equation (2)) from 232 °C to 547 °C, which was consistent with the results obtained by the XPS test. Additionally, the reaction to form Si–OH also proceeds without oxygen participating in the reaction to form CO (Equation (4)). In stage III, a part of the hydroxyl groups was oxidized to produce CO_2_. Additionally, hydroxyl condensation occurred in all parts of the silica aerogels, forming to the stable Si–O–Si bonds (Equation (6)) from 283 °C to 547 °C. The formation of Si–O–Si was conducive to the improvement of three-dimensional network structure of silica aerogel. The final stage, stage IV started after 547 °C with no chemical reaction, and all the primary particles gradually fused into secondary particles and sintered to form clusters. The aperture and the thermal conductivity continued to increase.

## 3. Numerical Simulation

**Figure 8:**
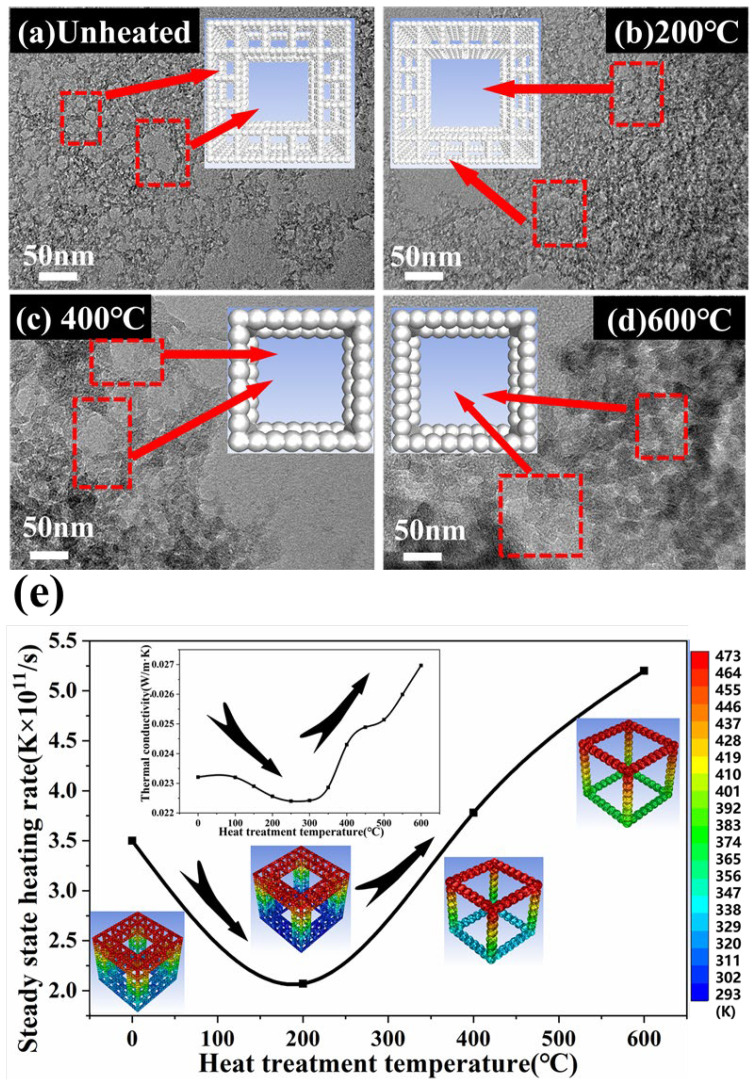
TEM images and corresponding structural models of the silica aerogel samples treated at different temperatures. (**a**) Unheated, (**b**) 200 °C, (**c**) 400 °C, (**d**) 600 °C. (**e**) Comparison of steady state heating rate and thermal conductivity trend of silica aerogel samples treated at different temperatures.

According to previous studies [33], silica aerogels have subtype characteristics within a certain scale. After heat treatment, the characteristic structure of silica aerogels has not undergone essential changes, so an appropriate fractal structure could be used to describe aerosolization. Based on the above analysis and previous study [34], combined the Intersecting Spheres structure and the classical Sierpinsky sponge, the Fractal-Intersecting Sphere models were established, as shown in Figure 8a–d and Figure S3. From the pore size distribution in Figure 3d, it could be concluded that with the heat treatment temperature increased in the process of pyrolysis, the main pore size of silica aerogel gradually increased and tended to be dispersed. Therefore, the effect of aperture was given priority in models. In the untreated silica aerogel, the primary particles were loosely bound, and the individuals were stacked into small clusters to form primary pores [35], which were generally below 10 nm. Small clusters accumulated to form larger pores, which were above 10 nm, and the sub-structures formed by the two types of pores shown in the models. As the heat treatment temperature increases, the primary particles were more closely combined, and the primary pores began to shrink. At the same time, the pores between the formation of small clusters gradually became larger. At 400 °C, most of the primary particles had been fused into secondary particles, making the fractal structure gradually disappeared. When it reached 600 °C, the primary particles continued to fuse, and the pore size continued to increase. Fluent software was used to simulate and solve the non-steady state energy conservation equation, as shown in the following formula (Equation (7)). The convection term of the energy equation was discretized in the QUICK format. In order to ensure the accuracy of the calculation, the equation convergence residual was specified below 10^−6^.

(7) $$\rho c\frac{\partial T}{\partial \tau }=\frac{\partial }{\partial x}\left(\lambda \frac{\partial T}{\partial x}\right)+\frac{\partial }{\partial y}\left(\lambda \frac{\partial T}{\partial y}\right)+\frac{\partial }{\partial z}\left(\lambda \frac{\partial T}{\partial z}\right)+\dot{\phi }$$

The model material was SiO_2_, the top temperature of the models was 473 K, and the bottom temperature was 293 K, which was adiabatic. 10–12 s and 10–10 s were chosen as the calculation time step and total time, respectively. According to the heating curve of the bottom surface, the heating rates of the steady-state area of the four models were selected for comparison. When the influence of the characteristic length of the model was removed, with the increase in heat treatment temperature (zero point means that the sample was untreated), the change trend of thermal conductivity was the same as the change trend of heating rate obtained from the model, as shown in Figure 8e. In the experiment, compared with the original samples, the thermal conductivity of the heat-treated samples can be reduced 3.49%, and with the increase in heat treatment temperature, the thermal conductivity increased 162.0% at 600 °C. In the simulation calculation, the steady state heating transfer rate could be as low as 93.32% of the original after 200 °C, and it could be increased by 2.19 times at 600 °C. One of the reasons was the removal of impurities to make the aerogel structure more uniform, and the other most important reason was the change of the fractal structure. It proved that the inference of the thermal conductivity caused by the structural change of silica aerogel was reasonable, and the model structure conformed to the actual situation.

## 4. Conclusions

In order to obtain the best thermal insulation performance of hydrophobic silica aerogel, a certain degree of heat treatment was performed on the sample. Analysis showed that as the heat treatment temperature rise, the thermal insulation performance of aerogel presents a trend of first increasing and then decreasing, where the thermal conductivity could be as low as 0.022 W/m·K at 250 °C. The pyrolysis process of aerogel is divided into four stages. In addition, inside the aerogel, because of lacking oxygen, the reaction would produce CH_4_. The form of Si–O–Si optimized the three-dimensional network structures to be beneficial to improve the thermal insulation performance of silica aerogel, and BET and pores volume could reach peak values of 993 m^2^/g and 4.75 cm^3^/g, respectively. It also promoted the fusion of aerogel primary particles to form secondary particles. The generation of internal gas also promoted the formation of macropores in the aerogel, which significantly improved the thermal conductivity of the aerogel. As a result, moderate heat treatment at 250 to 300 °C would maximize the thermal insulation performance of hydrophobic aerogel though optimizing its pore structure.

## 5. Experimental Methodology

Sample preparation. Hydrophobic aerogel with excellent performance was successfully synthesized by the sol-gel route under normal pressure (Figure 1). In this work, tetraethyl orthosilicate (TEOS, Sigma-Aldrich, St. Louis, MO, USA) was selected as the precursor. Other reagents including hydrochloric acid (37 wt%), ammonia (27 wt%), ethanol, and hexamethyldisiloxane were purchased form SCRC (Shanghai, China). Firstly, HCl was added to the hydrolysis solution of TEOS in EtOH to adjust the PH value to 3, with vigorously stirring to hydrolyze for 4 h. Then, NH_4_OH was added to the sol to obtain the gel when the pH was 6. The acidic and basic catalysts used were 0.1 mol L^−1^ HCl (aq) and 0.5 mol L^−1^ NH_4_OH (aq). The molar ratio of TEOS, EtOH, HCl, H_2_O, and NH_4_OH was 1:9.1:2.2 × 10^−3^:2.5:9.5 × 10^−3^. After aging for 8 h, The shredded gel was subjected to surface modification with 34% HMDSO/EtOH under 60 °C in a water bath, and the process was carried out under strong acid conditions. After drying under normal pressure, hydrophobic silica aerogel could be obtained. The silica aerogel reaches the preset temperature value (200 °C, 400 °C, 600 °C, 800 °C, and 1000 °C) with heating rate of 10 °C per minute in a muffle furnace and keeps it for two hours.

The apparent density of the silica aerogel could be calculated by the following formula [36]:

(8) $$\rho_{a}=\frac{m}{m_{1}-m_{2}+m_{3}}\rho H_{2}O$$

where $\rho_{a}$ is the density of SiO_2_ aerogel, kg/m^3^; and ρ is the density of distilled water. 

The porosity of the aerogels was obtained using the following formula [35]:

(9) $$porosity=\left(1-\frac{\rho_{a}}{\rho_{s}}\right)\times 100\%$$

where $\rho_{s}$ is the skeleton density of silica aerogel, considered as 2200 kg/m^3^ [37].

The water contact angle was measured by the contact angle goniometer SL200KS (Kino, Boston, MA, USA). The thermal conductivity of the silica aerogel was measured using the TC3000E instrument (Xiaxi Technology, Xian, China) using transient hotwire method. TG-IR analysis was performed by TL-9000 (Perkin Elmer, Waltham, MA, USA) instrument heated from 30 °C to 1000 °C with 10 K/min. FT-IR spectroscopy in this study was Nicolet 8700 (Thermo Nicolet, Waltham, MA, USA) instrument with the range of 400–4000 cm^−1^ to study the surface chemical modification of the samples. Morphology of samples was analyzed using a high-resolution field emission GeminiSEM 500 (ZEISS, Oberkochen, Germany). The pore size distribution (PSD), surface area and pore parameters of heated samples and virgin material were measured by the BET and BJH methods (Tristar II 3020M, Atlanta, GA, USA). The information regarding the functional group containing hydrogen of the samples was provided for the solid ^1^H NMR, and was conducted using a Bruker AVANCE AV400 (Bruker, Fallanden, Switzerland). The Powder X-ray diffraction (XRD) were measured with the angle of 10–80° by X’Pert MPD (PHILIPS, Amsterdam, The Netherlands) and the sample powder were distributed on a tap before the measurement. The information about the functional group and elemental composition of the silica aerogels can be obtained from the X-ray photoelectron spectroscopy (XPS), using an ESCALAB 250Xi (TermoFisher, Waltham, MA, USA). 

At the combustion beamline (BL03U) of the National Synchrotron Radiation Laboratory in Hefei, China, the synchrotron vacuum ultraviolet photoionization mass spectrometry (SVUV-PIMS) analysis was performed. In this work, aerogel silica samples were heated in the reactor from 20 to 800 °C by 15 °C/min. Nitrogen was used as the carrier gas to provide an inert atmosphere, which can introduce the pyrolytic vapor to the ionization chamber, in which the sampling capillary and synchrotron vacuum ultraviolet (SVUV) light meet at a perpendicular. The pyrolysis products were ionized by the crossed SVUV light, and the generated ions would be analyzed by reflection electron microscope TOF-MS. The test principle is shown in Figure 5. More information on this end station and beamline can refer to the relevant reference [38,39]. In order to verify the analysis of the structural changes caused by the changes in the surface groups of the silica aerogel after heat treatment, models were established by Fluent software to calculate the heat transfer based on the test results of the TEM and BET void structure, and then compared with the experimental data.

## Figures and Tables

**Table 1:** Thermal properties of the silica aerogel samples.

Sample	Bulk Density (g/cm^3^)	Thermal Conductivity (W/m·K)
Unheated	0.109	0.02321
150 °C	0.107	0.02290
200 °C	0.104	0.02256
250 °C	0.101	0.02240
300 °C	0.102	0.02242
350 °C	0.107	0.02286
400 °C	0.113	0.02430
450 °C	0.115	0.02489
500 °C	0.118	0.02514
550 °C	0.120	0.02599
600 °C	0.130	0.02697
800 °C	0.311	0.08450
1000 °C	0.497	0.12185

**Table 2:** BET surface area and pores volume of the silica aerogel samples.

Sample	BET Surface Area (m^2^/g)	Total Volume in Pores (cm^3^/g)
Unheated	878	3.23
200 °C	889	3.55
400 °C	993	4.75
600 °C	667	3.76
800 °C	508	2.78
1000 °C	54	0.23

**Table 3:** Compositions of the silica aerogel samples calculated by XPS results.

Sample	Si (%)	O (%)	C (%)	Si/O	Si/C
Unheated	22.88	44.28	32.84	0.52	0.70
200 °C	23.47	44.51	32.02	0.53	0.73
400 °C	20.72	51.42	27.86	0.40	0.74
600 °C	22.10	52.27	25.63	0.42	0.86
800 °C	25.92	58.47	15.61	0.44	1.66
1000 °C	29.96	64.07	5.97	0.47	5.02

## Data Availability

The data presented in this study are available on request from the corresponding author.

## Supplementary Materials

The following supporting information can be downloaded at: https://www.mdpi.com/article/10.3390/gels8030141/s1. Figure S1: Photographs of silica aerogel after heat treatment at different temperatures; Figure S2: N_2_ desorption–adsorption isotherms of the silica aerogel samples heated at different temperatures; Figure S3: Microscopic models of silica aerogel treated at different temperatures. Videos S1–S4: Heating process of silica aerogel sample treated at different temperatures.
