# Supplementary material for: Improvement of the Thermal Insulation Performance of Silica Aerogel by Proper Heat Treatment: Microporous Structures Changes and Pyrolysis Mechanism
Source: Gels. 2022 Feb 23;8(3):141. doi: 10.3390/gels8030141 (PMC8953605; doi:10.3390/gels8030141)
Supplement: Supplementary file 1 [file gels-08-00141-s001.zip › supplementary figures.pdf]

gels

Supplementary Materials

Improvement of the thermal insulation performance of silica aerogel by proper heat treatment: microporous structures changes and pyrolysis mechanism

Zhiyi Lun [1], Lunlun Gong [1], Zhongxin Zhang [1], Yurui Deng [1], Yong Zhou [1], Yuelei Pan [1,*] and Xudong Cheng [1,*]

State Key Laboratory of Fire Science, University of Science and Technology of China, Hefei, Anhui 230027, PR China

The file includes **Figures. S1–S3** and **Video S1–S4**.

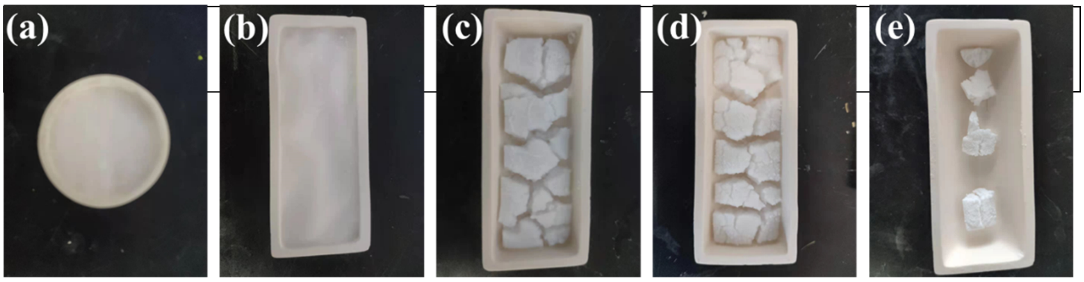

Figure S1. photographs of silica aerogel after heat treatment at different temperatures (**a**) untreated, (**b**) 200 °C, (**c**) 400 °C, (**d**) 600 °C, (e) 800 °C.

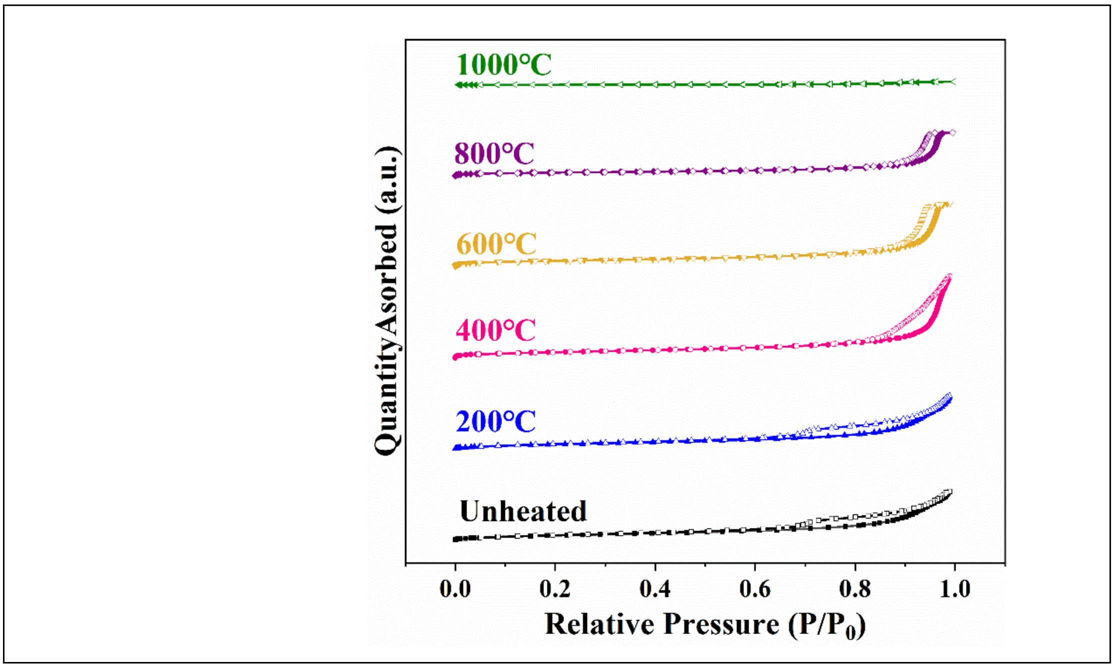

Figure S2. N_2 desorption-adsorption isotherms of the silica aerogel samples heated at different temperatures.

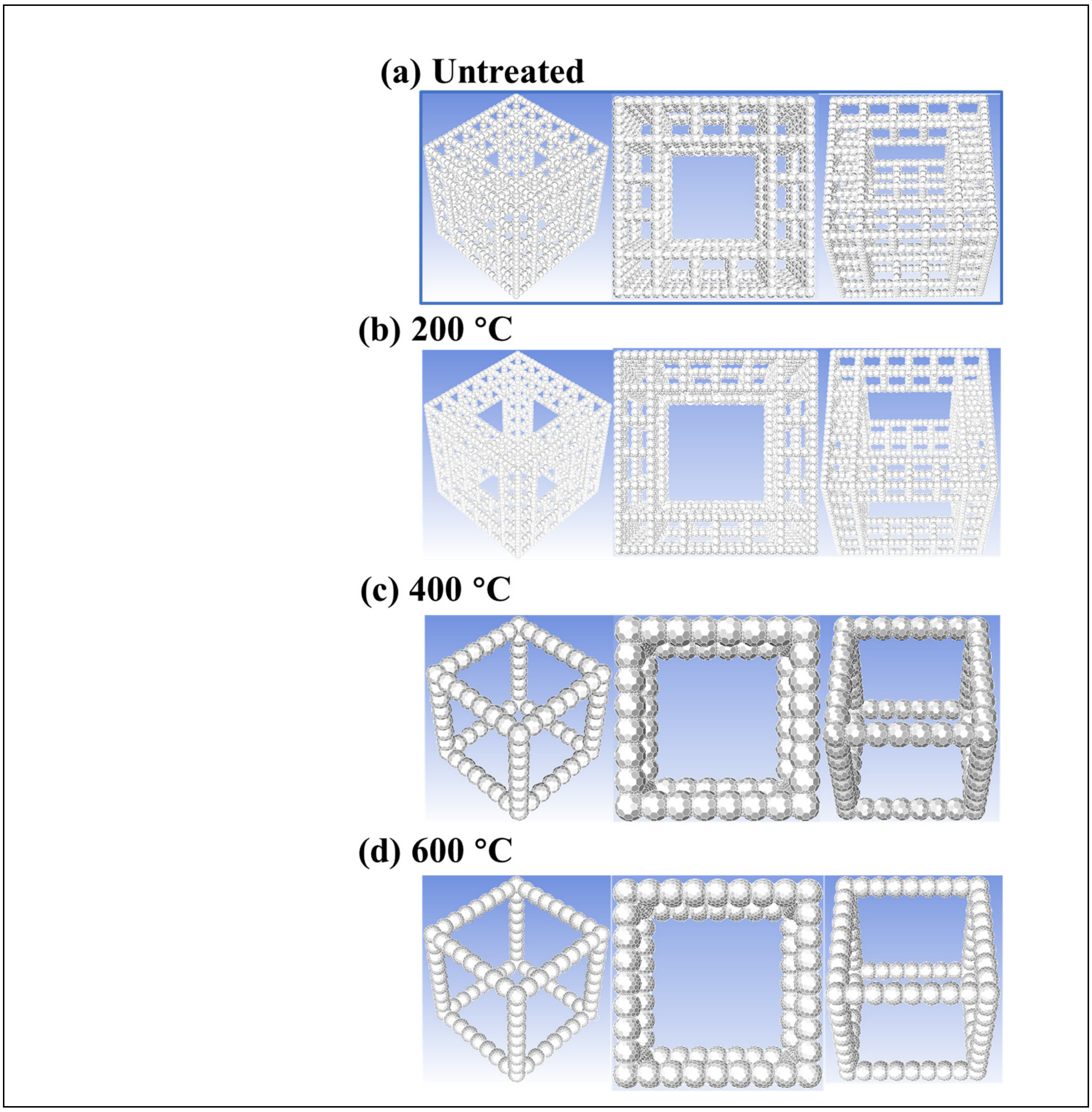

Figure S3. Microscopic models of silica aerogel treated at different temperatures (**a**) untreated, (**b**) 200 °C, (**c**) 400 °C and (**d**) 600 °C.

Supporting Video S1-S4: Heating process of silica aerogel sample treated at different temperatures (untreated, 200 °C, 400 °C and 600 °C).
